# IP_3_Rs puff along: A SNAPpy dance with IP_3_ and Ca^2+^

**DOI:** 10.1016/j.jbc.2023.103010

**Published:** 2023-02-10

**Authors:** Arya Y. Nakhe, David A. Jacobson

**Affiliations:** Department of Molecular Physiology and Biophysics, Vanderbilt University, Nashville, Tennessee, USA

## Abstract

Concerted openings of clustered inositol 1,4,5-trisphosphate receptors (IP_3_Rs) result in short, localized Ca^2+^ bursts, also called puffs, which are crucial regulators of Ca^2+^-dependent signaling processes. However, the processes regulating Ca^2+^ puff amplitude (average ∼0.5 ΔF/F_0_) and duration (at half-maximal; average ∼25-30 ms) have yet to be elucidated. A recent study in JBC by Smith and Taylor determined that Ca^2+^ puff amplitude is independent of IP_3_R cluster density and that the termination of IP_3_R Ca^2+^ puff is regulated by IP_3_ dissociation, illuminating the steps of this regulatory dance.

Inositol triphosphate (IP_3_) generated by phospholipase C–mediated cleavage of phospholipids activates endoplasmic reticulum (ER) Ca^2+^ release through IP_3_ receptors (IP_3_Rs), affecting downstream processes such as gene transcription, mitochondrial respiration, hormone secretion, and electrical excitability. IP_3_R activity is finely tuned not only by mechanisms that control IP_3_ production but also by other molecules and proteins that bind to and regulate IP_3_Rs (1). For example, IP_3_Rs are coactivated by IP_3_ and nanomolar concentrations of Ca^2+^, potentiated by ATP, and inhibited by high concentrations of Ca^2+^. IP_3_R activity is also modulated by a multitude of interacting partners some of which also regulate their distribution, IP_3_ and Ca^2+^ sensitivity, and the kinetics of Ca^2+^ release. Additionally, redox state as well as covalent modifications such as phosphorylation affect IP_3_R activity (1). The recent near-atomic level structures of IP_3_Rs have begun to uncover regions of the protein involved in molecular interactions and conformational changes that affect activity (2, 3). Thus, advanced biochemical and physiological studies are beginning to correlate IP_3_R structure with function.

Clear identification of IP_3_R ER Ca^2+^ release has recently been achieved with total internal reflection fluorescence microscopy, which allows optical patch clamp measurements of discrete IP_3_R clusters (4). These localized and brief “Ca^2+^ puffs” result from regenerative propagation of Ca^2+^ signals due to IP_3_- and Ca^2+^-mediated openings of multiple IP_3_Rs in a clustered nanodomain (∼6 receptors open per Ca^2+^ puff from a cluster). Upon IP_3_ binding, Ca^2+^ that is first released by the IP_3_R binds to high affinity Ca^2+^-binding sites in neighboring IP_3_Rs, thus amplifying the rising phase of the Ca^2+^ puff. As Ca^2+^ concentration increases during the rising phase of Ca^2+^ puffs, it binds to low affinity site in the IP_3_R, inactivating further Ca^2+^ release. This process can be reinitiated from the same cluster following IP_3_R recovery from inactivation or activation of IP_3_Rs in the cluster that never underwent Ca^2+^-dependent inactivation. This regenerative process is further tuned by IP_3_ abundance, which correlates with Ca^2+^ puff frequency. Although many studies have characterized the kinetics of IP_3_R-mediated Ca^2+^ puffs, the mechanisms which control their amplitude and termination have not been determined.

A recent study in the JBC by Smith and Taylor utilized tetracycline-inducible expression of human IP_3_R3, harboring an N-terminal SNAP-tag (SNAP-IP_3_R3) to assess Ca^2+^ puff amplitude and termination (5). They monitored IP_3_R3 expression and ER distribution with total internal reflection fluorescence microscopy following fluorescent labeling of the IP_3_R3 SNAP-tag. They also analyzed Ca^2+^ puffs from IP_3_R3 puncta with a Ca^2+^ dye in response to flash photolysis of a caged analog of IP_3_ (ci-IP_3_); this not only allowed rapid release of IP_3_ but also a tight control of total IP_3_ concentration. Using this approach, Smith and Taylor were able to determine a) how IP_3_R3 expression affects receptor localization, b) how IP_3_R3 density in a microdomain influences the amplitude and frequency of Ca^2+^ puffs, and c) how IP_3_ dissociation impacts Ca^2+^ puff termination (Fig. 1).

**Figure 1:**
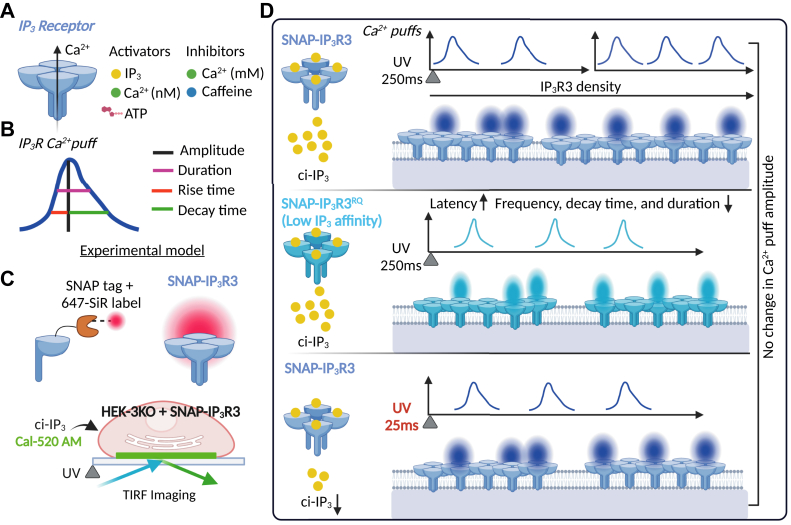
**IP**_**3**_**R Ca**^**2+**^**puff termination is controlled by IP**_**3**_**dissociation rate as determined by Smith and Taylor.***A*, molecular regulators of IP_3_Rs. *B*, representative Ca^2+^ puff waveform detailing parameters of puff kinetics analyzed in the study by Smith and Taylor. *C*, illustration of the TIRF microscopy approach utilized to monitor Ca^2+^ puffs from HEK cells expressing SNAP-tagged IP_3_R3 labeled with a far-red dye. *D*, Smith and Taylor determined that IP_3_R3 Ca^2+^ puff frequency increases with receptor density. In IP_3_R3s with reduced affinity for IP_3_ or at low IP_3_ concentration, Ca^2+^ puff amplitude remains equivalent; however, puff frequency and duration are reduced. IP_3_R, inositol 1,4,5-trisphosphate receptor; TIRF, total internal reflection fluorescence.

The authors determined that ER-localized Ca^2+^ puff frequency and amplitude correlated with IP_3_R3 density. IP_3_R3 expression positively correlated with IP_3_R cluster density, IP_3_R Ca^2+^ puff frequency, and total number of IP_3_R3 microdomains. Moreover, IP_3_ generation resulted in faster Ca^2+^ puff initiation from microdomains with higher IP_3_R3 density. These findings are consistent with previous reports showing Ca^2+^ puff frequency is positively correlated with IP_3_ concentration (6). Interestingly, they show that IP_3_R3 expression level did not affect many properties of Ca^2+^ puffs, including amplitude, rise time, decay time, and duration; this indicated that during each Ca^2+^ puff, the number of open IP_3_R3s in a microdomain remains constant irrespective of IP_3_R3 density. As ER Ca^2+^ release is critical for cellular signaling, IP_3_R Ca^2+^ puff amplitude (*e.g.*, Ca^2+^ signal intensity) is likely capped by microdomain-dependent regulation of IP_3_Rs. This regulation was suggested by Smith and Taylor to be dependent on both interactions between IP_3_Rs, limiting the number of open IP_3_Rs, and Ca^2+^-dependent IP_3_R inactivation during the rising phase of Ca^2+^ puffs. This is supported by previous data showing that rapid Ca^2+^ buffering increases the number of active IP_3_Rs in a microdomain (7).

The authors also examined if dissociation of IP_3_ from IP_3_R3s was involved in Ca^2+^ puff termination. This was assessed using IP_3_R3s with normal or lower IP_3_-binding affinity (IP_3_R3^R568Q^). Although IP_3_R3^R568Q^ and WT IP_3_R3 show similar Ca^2+^ puff amplitude, IP_3_R3s with lower IP_3_ affinity showed reduced Ca^2+^ puff frequency, shorter duration, faster decay time, and increased latency to puff initiation. Thus, reducing IP_3_R affinity for IP_3_ results in faster termination of Ca^2+^ puffs. This supports previous work showing that IP_3_R Ca^2+^ puff duration correlated with IP_3_ affinity of IP_3_R subtypes (IP_3_R2>IP_3_R1>IP_3_R3) (6). Interestingly, Ca^2+^ puff frequency was equivalent between IP_3_R3^R568Q^ and IP_3_R3 in response to high and low IP_3_ concentrations, respectively. This suggested that steady-state IP_3_ occupancy of IP_3_Rs is a determinant of Ca^2+^ puff termination. Although multiple IP_3_Rs are active during each Ca^2+^ puff, Ca^2+^ puff termination occurs in a rapid concerted manner. Therefore, while Smith and Taylor have clearly shown that Ca^2+^ puff termination involves IP_3_ release, the coordination of this process among multiple IP_3_Rs is likely more complex (*e.g.*, allosteric modulation).

Using controlled IP_3_R3s expression and ligand concentration, Smith and Taylor illuminated how IP_3_R3 density and IP_3_ dissociation influence Ca^2+^ puff frequency, amplitude, and termination. However, in physiological systems, IP_3_Rs function in a complex during conditions of graded molecular interactions and posttranslational modification. Thus, studies examining IP_3_R-mediated Ca^2+^ puff kinetics with more intact cellular milieu that are beginning to emerge will help to delineate physiological IP_3_R function. For example, Kras-induced actin-binding protein interaction with IP_3_R has recently been shown to regulate the amplitude of Ca^2+^ puffs and the number of active IP_3_Rs within a cluster (8). Additionally, ER localization-dependent modulation of IP_3_R function requires further investigation, such as Ca^2+^ release from IP_3_Rs recruited to mitochondria-associated membranes.

This study illuminates the conserved and carefully choreographed spatio-temporal dance of microdomain Ca^2+^ release from ER-localized channels, which highlights the importance of these cellular signaling modalities. However, the mechanisms involved in the precise control of Ca^2+^ puff amplitude and duration remain to be determined. Interestingly, although IP_3_Rs and ryanodine receptors (RyRs) are activated by different intracellular signals, the independence of Ca^2+^ release rise time and amplitude on receptor cluster density is maintained in both channels (5, 9). This might be due to the similarities in Ca^2+^-induced closure (inactivation) of these receptors, which would likely cap Ca^2+^ puffs/sparks at an equivalent amplitude even in microdomains with a high receptor density (10). Additionally, the number of active RyRs and IP_3_Rs in a microdomain is also constrained by inter-receptor interactions and/or other allosteric pathways. Although Ca^2+^ release during puffs/sparks is tightly regulated in the microdomain, puff/spark initiation and frequency correlate with receptor density (5, 9). These similarities between IP_3_R and RyR regulation underlie the importance of Ca^2+^ puffs/sparks to cellular function. The findings by Smith and Taylor take a significant step towards fully elucidating the complex and coordinated regulation of endogenous IP_3_Rs during Ca^2+^ puffs.

## Conflict of interest

The authors declare that they have no conflicts of interest with the contents of this article.
